# Preharvest supplemental lighting enhances productivity and modulates postharvest quality in hydroponically grown Apulian unripe melon ‘Scopatizzo’ ( *Cucumis melo* L.)

**DOI:** 10.1002/jsfa.70792

**Published:** 2026-06-10

**Authors:** Onofrio Davide Palmitessa, Marica Troilo, Carlo Porfido, Roberto Terzano, Davide De Angelis, Massimiliano Renna, Francesco Caponio, Pietro Santamaria

**Affiliations:** ^1^ Department of Soil, Plant and Food Sciences University of Bari ‘Aldo Moro’ Bari Italy

**Keywords:** agro‐biodiversity, cold storage quality, landraces, shelf‐life, sustainable vegetable production

## Abstract

**BACKGROUND:**

Preharvest light management is increasingly used to enhance yield and fruit quality in soilless systems, yet its effects on unripe melon fruits remain poorly understood. This study evaluated the impact of supplemental red–blue light‐emitting diode (LED) lighting applied during cultivation in a closed‐cycle hydroponic system on productivity, mineral composition and postharvest behaviour (4 °C for up to 17 days) of the Apulian melon landrace ‘Scopatizzo’ (*Cucumis melo* L.), consumed as an unripe fruit alternative to cucumber (*Cucumis sativus* L.).

**RESULTS:**

Principal component analysis highlighted a clear separation between cucumber and ‘Scopatizzo’ samples. In ‘Scopatizzo’, LED supplementation increased fruit number per plant by approximately 29% (14 *versus* 11 fruits per plant) without affecting fruit mineral composition at harvest. During cold storage, LED‐grown fruits maintained approximately 18% higher firmness at the end of storage than control fruits. Variations in moisture content, pH and titratable acidity were mainly driven by storage time and were unaffected by the light regime. LED‐grown fruits showed up to 12% higher chlorophyll and 28% higher glucose concentrations at harvest than controls, whereas total phenolics and carotenoids increased during storage in both treatments (+80% and +22%, respectively), indicating a predominant effect of cold‐induced metabolic responses during postharvest.

**CONCLUSION:**

Preharvest LED lighting enhanced productivity and improved specific postharvest quality traits of ‘Scopatizzo’ without altering its mineral profile, supporting the integration of light management and soilless cultivation to valorise melon landraces and extend their marketability as fresh, whole‐edible products. © 2026 The Author(s). *Journal of the Science of Food and Agriculture* published by John Wiley & Sons Ltd on behalf of Society of Chemical Industry.

## INTRODUCTION

‘Scopatizzo’, belonging to *Cucumis melo* L., is a local melon variety grown in Apulia region (southern Italy), whose fruits are picked while still immature, and meant to be eaten fresh in salads or plain, serving as a favourable alternative to cucumber (*C. sativus* L.) because of its superior sensory and digestive profile, higher content of bioactive compounds such as polyphenols and tocopherols, and the absence of cucurbitacins, which are responsible for bitterness in cucurbit fruits.^1^, ^2^ As a result, this product is widely consumed and has recently gained increasing market recognition beyond its area of origin, including northern European markets, thereby raising specific interest in its postharvest behaviour. Specifically, these melon fruits are harvested before the seeds are fully defined and are marketed as ‘whole‐edible’ fruits, meaning that consumers enjoy not just the mesocarp but also the exocarp and placental portions. From a nutritional point of view, the fact that the entire fruit can be consumed is relevant, particularly because previous studies reported that placenta is rich in polyphenols and tocopherols, most notably *α*‐tocopherol, which are recognised as functional compounds associated with cardiovascular benefits and contribute to the antioxidant intake provided by fresh vegetables.^3^, ^4^ Therefore, whole‐edible immature melon fruits may represent an interesting dietary source of bioactive and antioxidant compounds. Recently, a population‐based and clinical study has demonstrated that ‘Scopatizzo’ fruits have superior sensory and digestive profiles over common cucumber.^1^ Interestingly, this local variety is recognised under the list of traditional agri‐food products of Puglia (TAP).^5^ This recognition applies to Italian products whose methods of processing, storage and ripening have been established and maintained for at least 25 years. Introduced over two decades ago within Italian legislation, the TAP designation serves as a useful framework for cataloguing traditional Italian food products. Additionally, this designation is familiar to the average consumer and can act as a stepping stone towards obtaining further certifications, including Geographical Indication (such as Protected Designation of Origin) and Traditional Specialities (TSG).^6^

Like other local Apulian varieties of unripe melon, ‘Scopatizzo’ is usually grown in open fields during the spring–summer season.^7^ However, in a previous study, Somma *et al*.^8^ demonstrated that the cultivation of this local variety in rockwool soilless systems under a supplemental light‐emitting diode (LED) light was effective for growing the plants outside the natural growing season, also obtaining an average fruit production of 15 kg plant^−1^ during 80 days after transplant crop cycle, comparable to that of commercial hybrids of cucumbers grown in the same conditions. Moreover, the rising interest in ‘Scopatizzo’ can also be attributed to its compatibility with the nutrient film technique (NFT) system,^9^ which enables cultivation using moderately saline water without affecting fruit yield or quality, making it a compelling alternative to traditional cucumber farming.^10^ Soilless cultivation techniques enable precise regulation of plant growth conditions, resulting in high productivity, improved crop quality and efficient utilisation of water and nutrients.^11^

Furthermore, while some authors^12^, ^13^ have reported data on the minimally processed ‘Barattiere’ (another Apulian variety of unripe melon), there are no studies reporting the postharvest performance of unripe melon whole fruits. To this end, it is important to highlight the growing interest of the international market in the fruits of local unripe melon varieties,^14^ which represent a valuable component of the rich plant biodiversity heritage of the Apulian region.

Considering all the above remarks, the main objectives of the study reported here were to evaluate the postharvest performance of the local unripe melon landrace ‘Scopatizzo’ grown in a closed‐cycle NFT system under two lighting regimes, and to determine how preharvest light conditions shape the physicochemical, structural and nutraceutical quality of the fruits during storage. Specifically, the study aimed to: (i) characterise the initial fruit quality attributes and mineral profile at harvest and their evolution during cold storage; (ii) assess whether LED lighting can enhance fruit firmness retention, pigment stability, sugar content and antioxidant properties during shelf‐life; and (iii) contextualise the postharvest behaviour of ‘Scopatizzo’ through comparison with cucumber (Babystar RZ F1 (22‐707), Rijk Zwaan), included as an external commercial reference.

## MATERIALS AND METHODS

### Greenhouse activity

#### Plant materials and growing conditions

The experimental trial was carried out at the experimental farm La Noria of the National Research Council, Institute of Sciences of Food Production (Mola di Bari, Italy; 41.062156° N, 17.066914° E) in an unheated polymethacrylate greenhouse with a maximum height of 4.5 m.

‘Scopatizzo’ local variety (*Cucumis melo* L.) was grown from September to November 2023. The seedlings were purchased from a local plant nursery and placed, at the phenological stage of two true leaves, in rockwool cubes (Grodan Delta, 75 × 75 × 65 mm^3^). At the phenological stage of four true leaves, the cubes were transferred to NFT cultivation modules, and the experimental trial started. Seven days after transplant (DAT) the main stem of each plant was tied to a string and raised vertically. Instead, in order to allow uniform flowering and fruiting, based on the work carried out by Somma *et al*.,^8^ the primary and secondary shoots were pruned after the second node.

#### Cultivation system and experimental treatments/design

The cultivation system used was a closed‐cycle NFT. Twenty plants per bench were grown on 11 aluminium benches (600 cm × 50 cm, length × width), with plants spaced 30 cm apart within each channel and the NFT channels spaced 100 cm apart (inter‐row distance), resulting in a plant density of 2.5 plants m^−2^. The benches were covered with a black (inner) and white (outer) film to avoid (i) direct contact between roots and aluminium benches and (ii) the sun radiation reaching the roots and the nutrient solution (NS), causing water evaporation. Each NFT bench was irrigated independently, and it represented the experimental unit, whereas fruits collected from each bench were considered sampling units for postharvest analyses. In order to allow the NS to flow evenly, each bench had a slope of 2% and a 90 L tank was placed at the base of the bench in which the NS consumed by the plants was re‐integrated, while the drainage NS was recovered and recirculated. A climate control unit inside the greenhouse allowed the recording of the following climate parameters: temperature, relative humidity, daily light integral (DLI) and CO_2_, while the total light integral (TLI) was calculated as the sum of DLIs. The first and the last benches were not considered for data collection to exclude border effects, while the remaining nine benches were used for the application of three experimental treatments with three repetitions. The experimental design used was the randomised block and the treatments applied were: (i) C (control: the plants were grown without the addition of supplementary light); (ii) L: plants were grown with the application of artificial lighting with LED technology with R + B spectrum, i.e. blue light (450 nm) + red light (660 nm). Two modules with the same light spectra (GreenHouse Interlight produced by C‐Led, Imola (BO), Italy), measuring 2490 × 110 × 57 mm^3^, were placed above the plants of six benches. Each module carried two series of LEDs with 120° luminous flux angle. Supplementary photosynthetic photon flux density (PPFD) was 170 μmol m^−2^ s^−1^ at a horizontal distance of 30 cm from the lamp. The light spectrum ratios were described by Palmitessa *et al*.^15^ The interlight modules were placed 25 cm below the apical meristem and the height was adjusted once a week to maintain a constant distance between the apical meristem and the fixtures, to ensure uniform light distribution and intensity. The light treatments started on 8 September with the LED modules above the apical meristems (the plants were 20 cm tall) and continued until the end of the crop cycle. The supplementary lighting was operated automatically by a system composed of a CR1000 datalogger (Campbell Scientific, Logan, UT, USA) and a quantum sensor (LI‐190R, LI‐COR, Lincoln, NE, USA) for 16 h day^−1^. The system calculated incoming sunlight in the greenhouse on a four‐second basis and turned on the LED light bars whenever the ambient PPFD dropped below 200 μmol m^−2^ s^−1^. The bars remained on until sunlight PPFD again rose above this set point.

#### NS composition and management

To facilitate NS uptake by the plants, and to avoid root anoxia phenomena, the NS was distributed at a flow rate of 0.9 m^3^ h^−1^. To promote the oxygenation of the NS, the fertigation schedule included the NS supply for 50 min per hour from 7 a.m. to 7 p.m., and subsequently 20 min fertigations were scheduled at 8 p.m., 9 p.m., 11 p.m., 3 a.m., 5 a.m. and 6 a.m., as there was no solar radiation.

A stock of NS was prepared in 7000 L tanks before the start of the experimental activity with the following macronutrient concentrations (mmol L^−1^): 10 N‐NO_3_, 0.45 N‐NH_4_, 6 K, 1.5 P, 1 Mg, 3.5 Ca, 2 S. The stock NS was used for the replenishment of the 90 L tanks placed at the base of each bench. Subsequently, every 2 days the consumed NS in every bench was replenished and its volume recorded. The NS consumption was used to calculate water use efficiency by dividing the NS consumption (L) by the fruit production (kg).

#### Fruit and growth analysis

**Figure 1:**
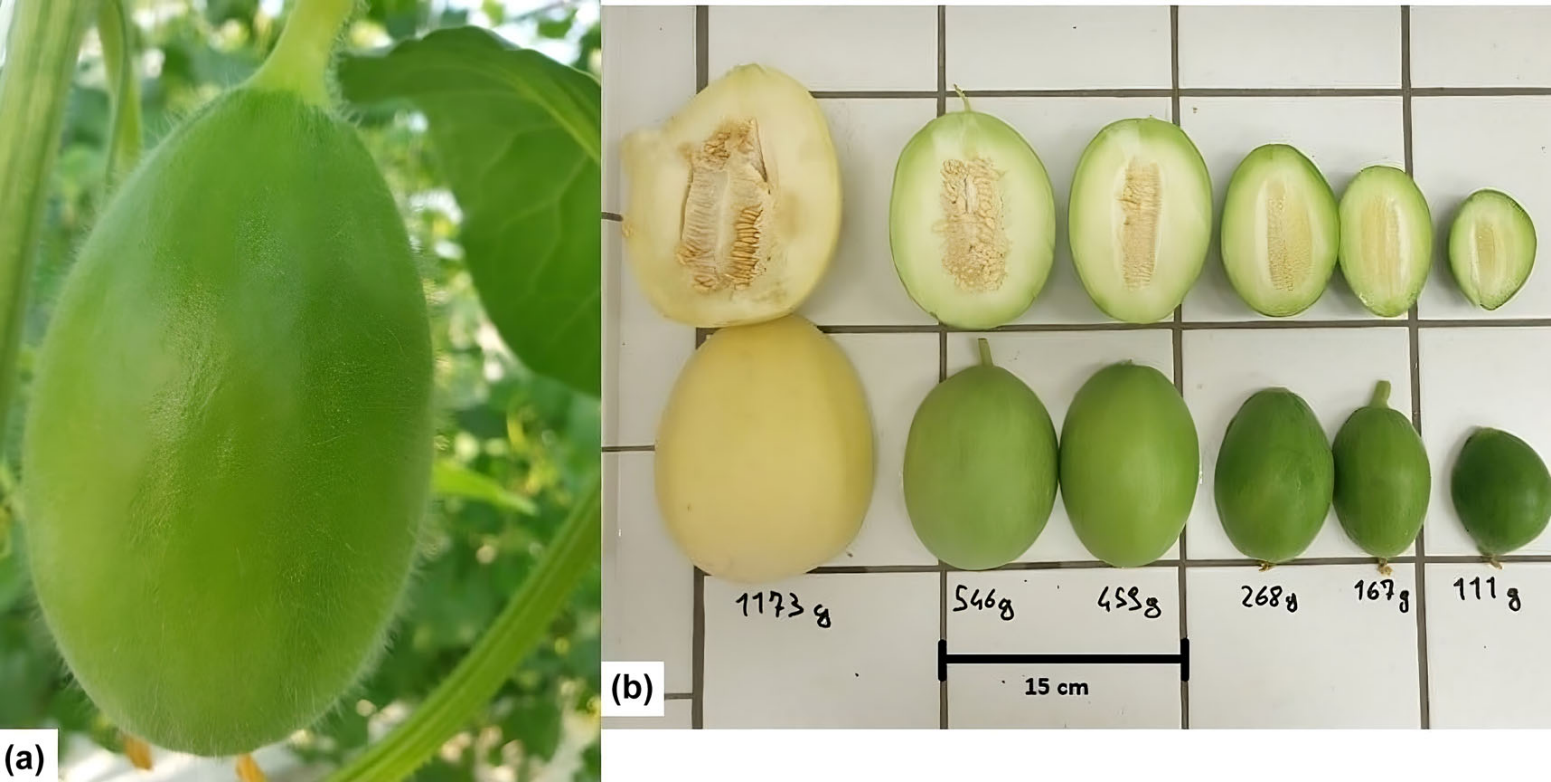
(a) Detail of the fruits of ‘Scopatizzo’ (*Cucumis melo* L.) harvested at a size of around 160–180 g, when the seeds were barely consumed and placenta was not clearly distinguishable from mesocarp (b).

The harvest of the fruits started 28 DAT, when the weight was approximately 160–180 g fruit^−1^ (Fig. 1) and it was performed every 23 days, depending on the actual size of the fruits. During each harvest operation, the number of harvested fruits was recorded, as well as the total fruit fresh weight (FW) and fruit dry matter (DM). DM was determined by placing the samples in a forced‐draft oven at 65 °C until constant weight.

#### Macro‐ and micronutrient analysis of fruits

The concentration of selected macronutrients (i.e. Mg, P, K, Ca) and micronutrients (i.e. B, Mn, Fe, Cu, Zn), along with that of Na, was determined using inductively coupled plasma optical emission spectrometry (5800 VDV, Agilent Technologies Inc., Santa Clara, CA, USA) in C and L fruit samples at 42 and 61 DAT. For such analysis, freeze‐dried ‘Scopatizzo’ samples were pulverised using a vibro‐milling system (MM 400, Retsch GmbH, Haan, Germany) and 150 mg aliquots were subjected to microwave‐assisted digestion (Multiwave GO, Anton Paar, Graz, Austria) in concentrated HNO_3_ (69%, TraceSELECT™, Honeywell Fluka) and H_2_O_2_ (30%, for trace analysis, Supelco). Finally, digested samples were diluted to 25 mL with Milli‐Q deionised water. All the analyses were performed in triplicate and the certified standard NIST™SRM™1573A (tomato leaves) was used as reference material to check the accuracy of the method.

### Postharvest assessment

#### Fruit cold storage and sampling pretreatment

Postharvest analyses were conducted to assess the effect of cold storage on the maintenance of physicochemical, sensory and nutritional characteristics of the fruits. The samples included ‘Scopatizzo’ grown under LED lighting (L) and under natural light (C) at 46 DAT and samples of cucumber (Babystar RZ F1 (22‐707), Rijk Zwaan; CTR), produced with a hydroponic system by an Apulian farm (F.lli Lapietra). Cucumber samples were included exclusively as an external commercial reference to contextualise the postharvest behaviour of ‘Scopatizzo’, considering the similar consumption pattern and commercial positioning of the two products. Fruits were stored at 4 °C for 17 days and analysed at T0 (harvest), T1 (4 days), T2 (8 days), T3 (12 days) and T4 (17 days) of refrigerated storage. Analyses were performed at each sampling time. Fruits were randomly collected from all biological replicates within each treatment at commercial maturity and subsequently distributed across different storage times.

#### Moisture, pH and titratable acidity determination

The moisture content was determined according to AOAC method 950.46 (Association of Official Analytical Chemists, 2006). The pH was measured using a digital pH meter (Edge HI2002, Hanna Instruments S.r.l., USA), after calibration with standard solutions at pH 4 and 7, according to AOAC method 981.12 (Association of Official Analytical Chemists, 1982). Titratable acidity values were determined as described by Palmitessa *et al*.^10^ Briefly, 2 g of homogenised pulp was mixed with 25 mL of Milli‐Q water and subsequently titrated using 0.1 N NaOH in the presence of phenolphthalein indicator. Results were expressed as grams of citric acid per 100 g of sample, according to the following calculation:

$$\text{Acidity}\left(\%\right)=\begin{array}{l} \frac{\mathrm{mL}\;\text{NaOH}\times 0.1N\times \text{equivalent weight of citric acid}}{\text{Weight of sample}\times 1000}\\ \times 100 \end{array}$$

Three measurements were performed for each sample at each time of storage.

#### Evaluation of texture and colour

The puncture test and texture profile analysis were performed on square pieces (2 × 2 × 2 cm^3^) of peeled fresh fruits using a texture analyser (Z1.0 TN, Zwick Roell, Ulm, Germany). For puncture tests, a flat‐headed stainless steel cylindrical probe (6 mm in diameter) and a 50 N load cell were used, at speed of 1 mm s^−1^, as described by Farcuh *et al*.^16^ Firmness (N) was evaluated at the end of the test, while the texture profile was assessed with a 36 mm diameter cylindrical probe and a 50 N load cell, modifying the method described in Bianchi *et al*.^17^ Two compression cycles were performed at 1 mm s^−1^ probe compression rate and 40% sample deformation, with 5 s pauses before the second compression. Springiness and chewiness were evaluated. Data were acquired using TestXPertII version 3.41 software (Zwick Roell, Ulm, Germany).

The colorimetric determination was carried out for both pulp and peel of ‘Scopatizzo’ samples, using a colorimeter (CM‐600d, Konica Minolta, Tokyo, Japan) supported by the software Spectramagic NX (Konica Minolta, Tokyo, Japan). Lightness (L*), redness (a*, i.e. the red–green coordinate) and yellowness (b*, i.e. the yellow–blue coordinate) were determined. The parameters were measured at several points internally and externally of samples. The white index (WI) of skin and pulp was calculated using the CIELAB values according to the following equation:

$$\mathrm{WI}=100-\sqrt{{\left(100-L^{*}\right)}^{2}+{\left(a^{*}\right)}^{2}+{\left(b^{*}\right)}^{2}}$$

For each sampling time, all measurements were conducted in triplicate.

#### Extraction and determination of bioactive compounds, pigments and antioxidant activity

For the determination of bioactive compounds and sugars, previously homogenised samples were freeze‐dried using a lyophiliser (BUCHI Italia s.r.l., Milan, Italy) set at −60 °C and 0.8 mbar.

The extraction of polyphenols and antioxidant compounds was carried out following the method described by Troilo *et al*.,^18^ with specific modifications. In detail, 1 g of sample was extracted using 6 mL of 80% methanol hydroalcoholic solution, subjected to an initial agitation phase for 10 min, followed by sonication (CEIA International S.A., 115/230 Vac 1–50/60 Hz–400VA max, Viciomaggio, Italy) for 20 min. Then the extract was shaken for an additional 20 min and centrifuged for 10 min at 10 000 × *g* at 4 °C using a Thump SL 16R Thermo Scientific centrifuge. The supernatants obtained were stored at −18 °C until further analysis. All extracts were prepared in duplicate.

The total phenolic content (TPC) was determined according to the Folin–Ciocalteu method as described by Troilo *et al*.^18^ Specifically, 980 μL of Milli‐Q water, 20 μL of extract and 100 μL of Folin–Ciocalteu reagent were mixed; after 3 min of incubation, 800 μL of 7.5% Na_2_CO_3_ was added and then incubated in the dark for 60 min. Absorbance was recorded with a Cary 60 spectrophotometer (Cernusco, Milan, Italy) at 720 nm. The results were expressed as milligrams of gallic acid equivalents (GAE) per gram of sample.

The antioxidant activity was assessed using the ABTS (2,2′‐azinobis(3‐ethylbenzothiazoline‐6‐sulfonic acid)) assay, after adding 50 μL of sample to 950 μL of ABTS solution. The absorbance was read at a wavelength of 734 nm after 8 min of incubation, and the results were expressed as μmol Trolox equivalents (TE) per gram of sample.

Chlorophyll and carotenoid contents were evaluated according to the spectrophotometric methods described by Pasqualone *et al*.^19^ and Paradiso *et al*.,^20^ respectively. For chlorophyll evaluation, 0.5 g of sample was homogenised with 15 mL of acetone and shaken for 15 min. Then, the quantification was performed by reading the absorbance at 661.4 and 644.8 nm, and the chlorophyll content (mg kg^−1^) was calculated as the sum of chlorophyll a and chlorophyll b as follows:

$$\text{Chla}\;\left(\mathrm{mg}\;{\mathrm{kg}}^{-1}\right):\left(11,24\times A661,4\right)-\left(2,04\times A644,8\right)$$

$$\text{Chlb}\;\left(\mathrm{mg}\;{\mathrm{kg}}^{-1}\right):\left(20.13\times A644,8\right)-\left(4.19\times A661.4\right)$$

where A661.4 and A644.8 are the absorbances at wavelengths of 661.4 and 644.8 nm, respectively. For the carotenoid concentration, instead, 1 g of sample was mixed with 5 mL of water‐saturated *n*‐butyl alcohol, shaken for 3 h in the dark and centrifuged for 5 min at 12 000 × *g* at 4 °C. Absorbance of the supernatant was read at 435.8 nm, and the total carotenoid content, expressed as milligrams of *β*‐carotene per kilogram of sample, was calculated according to the following equation:

$$\text{Total carotenoid content}\;\left(\mathrm{mg}\;{\mathrm{kg}}^{-1}\right)=\frac{\mathrm{Abs}435.8\times V\;\left(\mathrm{mL}\right)\times 1000}{1.6632\times 100\times p\;\left(\mathrm{mg}\right)}$$

where Abs435.8 is the absorbance at 435.8 nm and 1.6632 is the extinction coefficient for a solution of 1 mg of *β*‐carotene in100 mL of water‐saturated *n*‐butyl alcohol, according to AACC method 14‐50.01.^21^

Glucose and fructose contents were determined according to Squeo *et al*.,^22^ with some modifications. Briefly, 1 g of sample was added to 10 mL of Milli‐Q water, shaken for 10 min and centrifuged for 5 min at 10 000 × *g* at 4 °C. The supernatant was filtered and injected into an HPLC‐RID system (1260 Infinity, Agilent Technologies Inc., Santa Clara, CA, USA) equipped with a cation‐exchange column (300 mm × 8 μm × 7.8 mm; Rezex™ RCM‐Monosaccharide Ca^2+^, Torrance, CA, USA). The analysis was carried out under isocratic conditions using water as mobile phase at a flow rate of 0.6 mL min^−1^, with column temperature set at 80 °C and refractometer at 35 °C. Identification and quantification were performed using glucose and fructose as external standards, and the results were expressed as mg g^−1^ of sample.

### Statistical analysis

For the greenhouse experiment, each NFT bench represented one biological replicate (experimental unit), resulting in three replicates per treatment. For postharvest analyses, fruits were randomly collected from all replicate benches within each treatment and distributed across storage times (T0–T4). At each storage time, fruits originating from all biological replicates were included in the analyses to ensure representative sampling of each treatment. The number of fruits analysed for each parameter is reported in the corresponding tables and figure captions. Unless otherwise specified, the experimental replicates used for statistical analyses corresponded to the three biological replicates represented by the NFT benches. Measurements performed in duplicate or triplicate within each biological replicate were considered technical replicates and were averaged prior to statistical analysis in order to reduce analytical variability. For the data collected during the greenhouse activity, depending on the dataset, one‐way or two‐way analysis of variance (ANOVA) was applied: (i) one‐way ANOVA when considering ‘Treatments’ as the only experimental factor; (ii) two‐way ANOVA when both ‘Treatments’ and ‘DAT’ were considered as fixed factors. For the data collected during the postharvest activities, two‐way ANOVA was applied: ‘Treatments’ and ‘Time of storage’ were considered as fixed factors. To determine differences between means, a Tukey test was used, with a significance level of 95%, using Minitab Statistical Software (Minitab Inc., State College, PA, USA). The dataset collected during the postharvest activities, including physicochemical, colorimetric and textural parameters, was explored using principal component analysis (PCA), computed with Minitab Statistical Software (Minitab 19, Minitab Inc., State College, PA, USA). Data (mean value of three sampling units) were subjected to autoscaling and mean centring before model computation.

## RESULTS AND DISCUSSION

### Greenhouse experiment

#### Yield and physiological parameters of ‘Scopatizzo’ plants

Although this study primarily focuses on postharvest performance, including preharvest data on plant productivity is essential, since postharvest physiology is strongly influenced by the growing conditions experienced by the fruits prior to harvest. Indeed, yield components, agronomic input management and the general physiological status of the plants determine the metabolic composition, firmness, water relations and structural characteristics of the fruits at harvest, which in turn shape their subsequent behaviour during storage. Including this information ensures that the postharvest results can be correctly interpreted, as it allows excluding possible confounding factors linked to preharvest stress, unbalanced vegetative growth or differences in fruit development among treatments.

From the date of transplant to the end of the culture cycle, 61 days had elapsed. In addition, for all treatments, fruit harvest began 28 DAT.

For the productivity of the ‘Scopatizzo’ plants, it is possible to verify how with supplementary LED lighting it increased by about 29% compared to control (Table 1). As can be seen from Table 1, the different productivity of the plants cannot be attributed to the different average weight of the fruits (which, as was described in the materials and methods section, were harvested at an average weight of 174.3 g), but to an increased number of fruits which, for the plants grown with supplemental radiation, amounted to 3.3 more fruits per plant than control (Table 1). In general, the DM content of the fruits was, on average, 4.47 g 100 g^−1^ FW and the total NS consumption was around 189 L plant^−1^ (Table 1). So, although the ‘Scopatizzo’ plants grown under supplemental lighting produced 29% more fruits than under natural light condition (Table 1), they did not uptake a higher amount of NS, resulting in a 26% increase in NS uptake efficiency compared with control (Table 1).

**Table 1:** Yield, fruit number, fruit FW and DM, NS consumption and water use efficiency (WUE) of ‘Scopatizzo’ (*Cucumis melo* L.) plants grown under natural light (C) and with artificial light (L)

	Yield (kg m^−2^)	Fruit number (n. plant^−1^)	Fruit FW (g fruit^−1^)	Fruit DM (g 100 g^−1^ FW)	NS consumption (L plant^−1^)	WUE (L kg^−1^ fruit)
Treatments						
C	4.80 ± 1.35	10.9 ± 2.0	177 ± 25	4.21 ± 0.22	195 ± 12	101 ± 12
L	6.20 ± 0.88	14.2 ± 2.1	175 ± 16	4.87 ± 0.34	188 ± 9	75 ± 8
Significance	*	*	ns	ns	ns	**

Significance: *P ≤ 0.05 and **P ≤ 0.01; ns, not significant. The values of yield and fruit number are mean ± SD of six samples (two plants for each repetition; *n* = 6); the values of fruit FW and fruit DM are mean ± SD of 20 samples (*n* = 20); and the values of NS consumption and WUE are mean ± SD of three samples (*n* = 3).

These results highlight the effectiveness of using supplemental lighting in protected environments to promote production of local genetic resources such as ‘Scopatizzo’, especially under conditions of limited seasonal light availability. The increase in fruit number per plant (Table 1), without affecting average fruit weight or DM content, supports the hypothesis that improved productivity is the result of an enhanced crop load rather than changes in individual fruit development (Table 1). This is particularly relevant when considering the potential for off‐season production of perishable, fresh‐market crops where high yields must be balanced with postharvest quality traits.^23^ Moreover, the improved NS uptake efficiency observed in the LED treatment is particularly noteworthy, as it implies a more sustainable input–output ratio, which is central to modern soilless systems designed to minimise environmental impact while maintaining product quality.^24^, ^25^ The observed productivity gains, in the absence of changes in vegetative growth, indicate a preferential allocation of assimilates towards reproductive organs. When artificial lighting is applied to increase DLI or extend the photoperiod, the plant produces a larger amount of carbohydrates. These additional photoassimilates increase the overall carbon supply available for transport within the plant, thereby providing more resources that can be partitioned among competing organs (sources → sinks^26^). This shift may positively influence fruit uniformity and the harvest index, both of which are closely linked to commercial value and shelf‐life.^27^ These findings lay the groundwork for the subsequent evaluation of postharvest behaviour. In fact, fruit quality and storage potential are not only determined by genetics and handling, but also strongly influenced by preharvest environmental conditions, including light and nutrient availability.^28^ By improving yield without compromising physiological stability or fruit density, supplemental lighting in soilless greenhouse cultivation appears to be a promising tool for enhancing the economic viability and market reach of underutilised cultivars such as ‘Scopatizzo’.

#### Macro‐ and micronutrient content in fruits

The mineral composition of ‘Scopatizzo’ fruits remained relatively stable across the two light treatments and between the sampling dates (Tables 2 and 3), suggesting that nutrient partitioning into the fruit was only marginally influenced by the growth conditions.

**Table 2:** Macronutrient content in fruits of ‘Scopatizzo’ (*Cucumis melo* L.) grown under natural light (C) and with artificial light (L), analysed at 42 and 61 DAT

DAT	Treatment	Mg	P	K	Ca
		mmol 100 g^−1^ DM			
42	C	22.5 ± 1.9	34.0 ± 3.7	104 ± 10.0	22.1 ± 3.0
	L	23.0 ± 1.7	32.5 ± 3.4	93 ± 9.0	23.2 ± 6.6
61	C	22.4 ± 0.8	33.0 ± 2.2	97 ± 5.0	25.3 ± 3.6
	L	22.7 ± 1.0	33.4 ± 1.8	99 ± 4.8	24.3 ± 1.8
Significance					
DAT		ns	ns	ns	ns
Treatment		ns	ns	ns	ns
DAT × Treatment		ns	ns	ns	ns

Significance: ns, not significant. Values are mean ± SD of the main factors: DAT (*n* = 6) and Treatment (*n* = 3).

**Table 3:** Micronutrient content in fruits of ‘Scopatizzo’ (*Cucumis melo* L.) grown under natural light (C) and with artificial light (L), analysed at 42 and 61 DAT

DAT	Treatment	Na	B	Cu	Fe	Mn	Zn
		mmol 100 g^−1^ DM					
42	C	7.31 ± 0.90	0.400 ± 0.001	0.020 ± 0.001	0.267 ± 0.035	0.056 ± 0.002	0.138 ± 0.007
	L	7.84 ± 1.40	0.402 ± 0.017	0.020 ± 0.001	0.196 ± 0.067	0.051 ± 0.001	0.132 ± 0.005
61	C	8.81 ± 0.08	0.385 ± 0.020	0.023 ± 0.005	0.275 ± 0.044	0.064 ± 0.009	0.145 ± 0.010
	L	8.81 ± 0.09	0.408 ± 0.018	0.023 ± 0.004	0.198 ± 0.068	0.068 ± 0.007	0.121 ± 0.009
Significance							
DAT		**	ns	ns	ns	ns	ns
Treatment		ns	ns	ns	ns	ns	ns
DAT × Treatment		ns	ns	ns	ns	ns	ns

Significance: **P ≤ 0.01; ns, not significant. Values are mean ± SD of the main factors: DAT (*n* = 6) and Treatment (*n* = 3).

Among macronutrients, the mean concentrations of Mg, P, K and Ca were 22.7, 33.2, 98.3 and 23.7 mmol 100 g^−1^ DM, respectively. Potassium was the predominant cation, representing more than 50% of total macronutrients, confirming its central role in osmotic regulation and fruit firmness, as previously observed in other *Cucumis melo* L. landraces.^2^, ^14^ The relative stability of these elements across treatments and harvest times indicates that ion uptake and allocation to the fruit tissues were already balanced before the onset of harvest and not substantially modified by the applied light regimes.

Among micronutrients, the average concentrations of B, Cu, Fe, Mn and Zn were 0.40, 0.02, 0.22, 0.06 and 0.13 mmol 100 g^−1^ DM, respectively (Table 3). These values fall within the physiological ranges reported for melons and other cucurbits,^2^ confirming an adequate micronutrient status of the fruits. Iron and boron were the most abundant trace elements, consistent with their roles in chloroplast functionality and cell wall stability, respectively.^29^, ^30^

The only relevant variation observed during the experiment concerned sodium (Na^+^), whose content increased by approximately 16% from 42 to 61 DAT. However, it is important to highlight that the Na^+^ concentrations measured remained well within ranges that do not pose any concern for consumers from a nutritional or food safety perspective.^31^ This trend is physiologically coherent: Na^+^ is not essential for mineral nutrition and tends to accumulate in the recirculating NS of closed‐cycle systems, progressively increasing its concentration in plant tissues as the crop cycle advances. In contrast, all other analysed macro‐ and micronutrients are essential elements actively taken up by the plants. Their concentration in the circulating NS typically decreases towards the end of the cultivation cycle due to continuous uptake and assimilation, which explains the stable mineral composition observed in the fruits across the two harvest dates (Tables 2 and 3). This rise may reflect a progressive accumulation in the fruit tissues due to transpiration flow reduction and concentration effects during maturation. Such behaviour is typical of hydroponically grown cucurbits under low drainage conditions, where Na uptake can increase in the late stages of growth.^32^

Overall, the mineral profile of ‘Scopatizzo’ fruits confirmed a balanced ionic composition largely unaffected by harvest timing or light quality. The stability of macro‐ and micronutrient contents under both lighting conditions suggests that, within the tested range, LED supplementation did not alter nutrient partitioning processes in the fruit, ensuring comparable nutritional quality to those produced under natural light.

Mineral composition was determined only at harvest time, to characterise the nutritional status of the fruits before storage. Mineral content is primarily defined by preharvest growing conditions and does not undergo meaningful changes during cold storage, since additional elements cannot be further supplemented once the fruit has been detached from the plant. The mineral profile at harvest allows a physiological interpretation of the subsequent postharvest behaviour, since elements such as K, Ca and Mg influence turgor regulation, membrane integrity, firmness retention and pigment stability, helping to explain the differences in physicochemical and structural parameters observed during shelf‐life.

### Postharvest evaluation

#### Visual inspection and exploratory analysis of data

**Figure 2:**
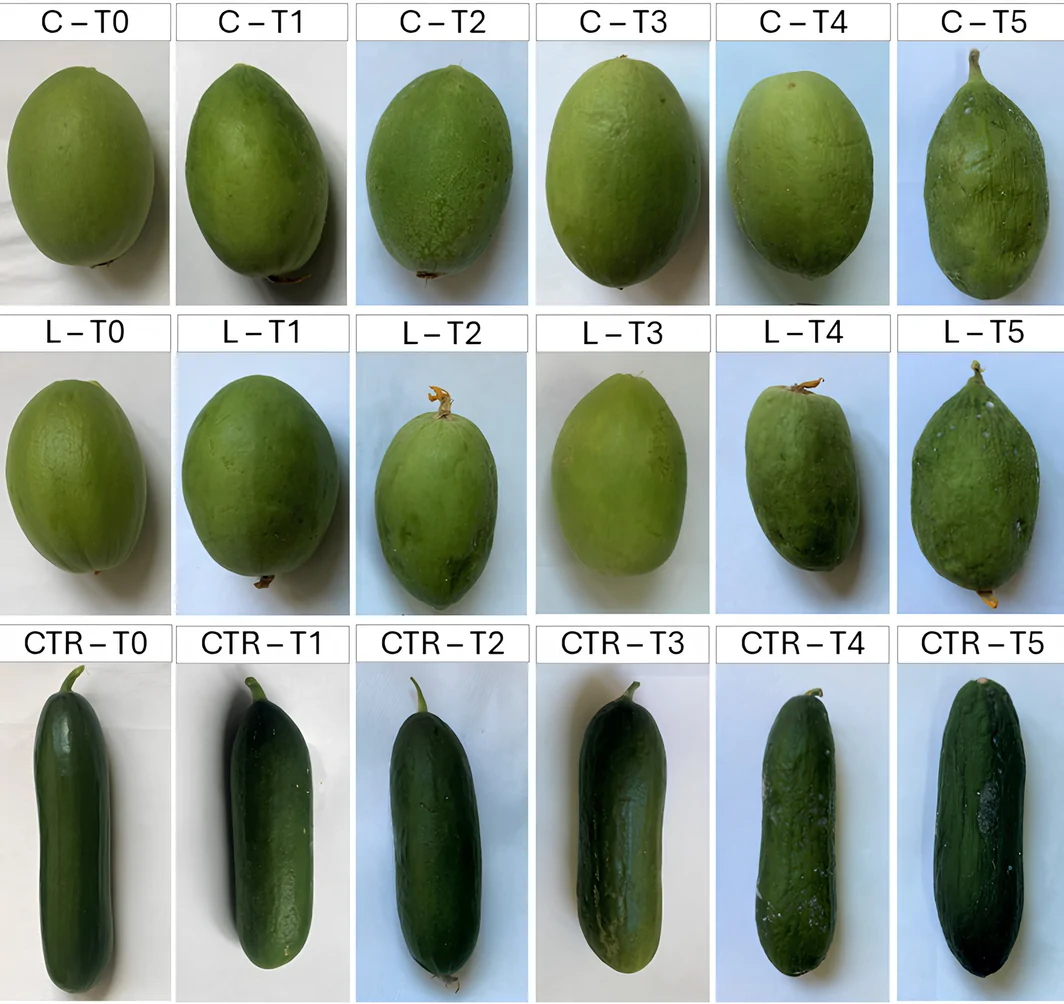
Appearance of ‘Scopatizzo’ (*Cucumis melo* L.) grown under natural light (C) and with artificial light (L), and samples of cucumber (*Cucumis sativus* L.; CTR). T0 = beginning of storage; T1 = 4 days of refrigerated storage; T2 = 8 days of refrigerated storage; T3 = 12 days of refrigerated storage; T4 = 17 days of refrigerated storage.

As shown in Fig. 2, at the beginning (T0) all samples appeared turgid, reflecting optimal postharvest quality. However, during storage, a progressive loss of firmness and freshness was observed, attributable to typical postharvest physiological processes such as transpiration and senescence, as well as to structural changes including modifications in polysaccharide composition of the cell walls and reduced cellular turgor, which are common in horticultural products.^16^, ^33^, ^34^, ^35^ As outlined in a previous section, cucumber was included as an external commercial reference, allowing the postharvest behaviour of ‘Scopatizzo’ to be interpreted within an analogous product category and providing a meaningful benchmark for shelf‐life comparison.

After 17 days of storage (T4), significant signs of deterioration were observed in cucumber samples (CTR), with visible mould growth on the surface, marking the limit of commercial acceptability. At the same time, ‘Scopatizzo’ samples remained commercially acceptable up to day 17 of storage. Beyond this period, the initial appearance of visible mould on the fruit surface was observed, indicating the loss of marketability.

These findings support the hypothesis that, in addition to its analogous use, ‘Scopatizzo’ shares a postharvest behaviour comparable to that of cucumber, allowing the commercial acceptability threshold for ‘Scopatizzo’ to be reasonably set at 17 days under the tested storage conditions.

**Figure 3:**
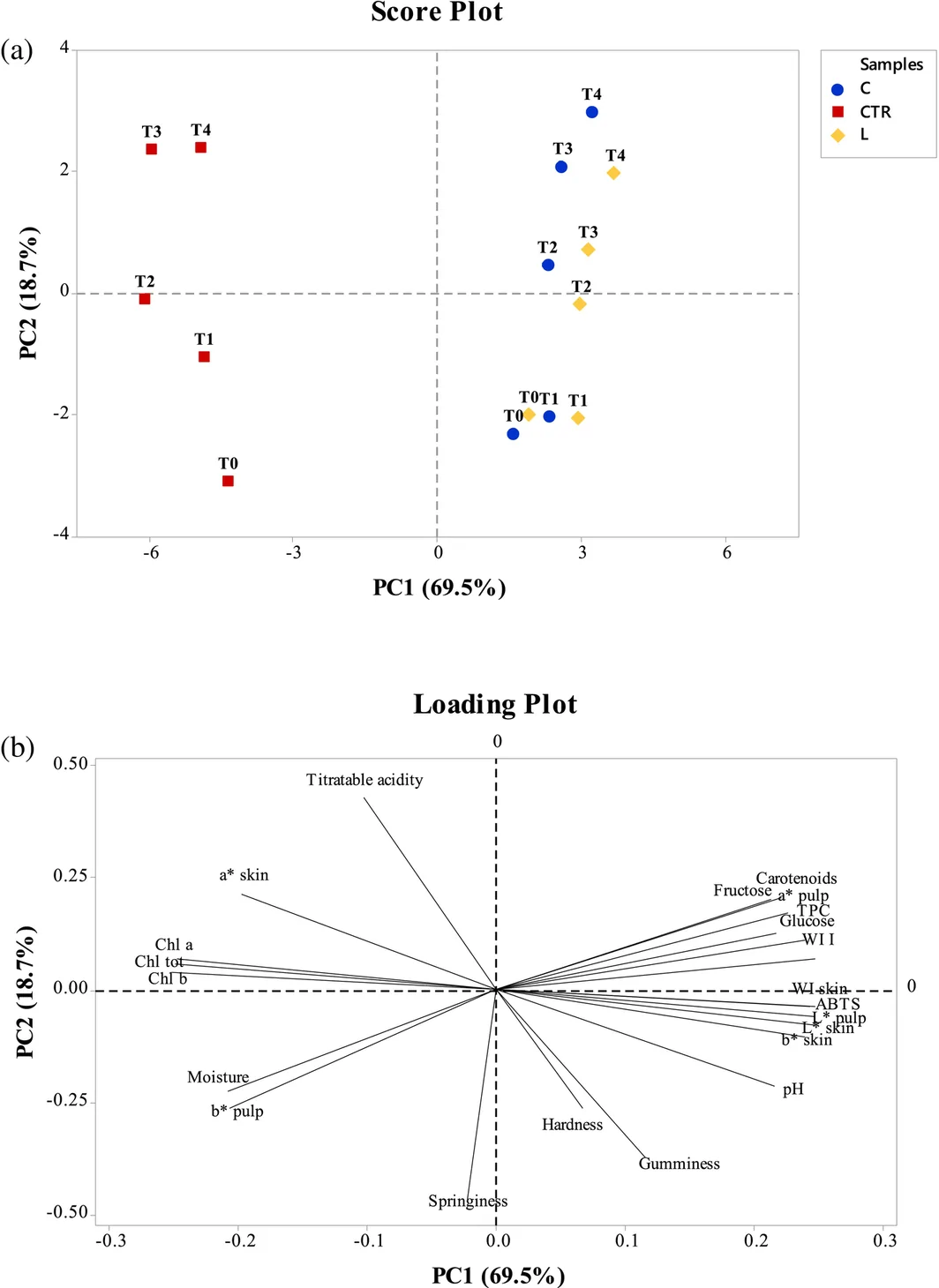
(a) Score plot and (b) loading plot related to time of storage and cultivation system of ‘Scopatizzo’ (*Cucumis melo* L.) grown with artificial light (L) and under natural light (C) and cucumber (*Cucumis sativus* L.; CTR).

Excluding the ‘Scopatizzo’ fruit mineral composition at harvest time, the entire dataset of postharvest analysis was subject to PCA. The score plot (Fig. 3(a)) reveals a clear distribution of samples along the first principal component (PC1), which explained 69.5% of the variance. Specifically, C and L showed positive scores on PC1, suggesting that differences between the two species were the main discriminating factor, exerting a greater influence than the cultivation system. PC2 (18.7% variability) reflets the effect of the storage time. Samples are distributed along this component according to the duration of cold storage, indicating that postharvest changes are effectively described by PC2. The loading plot (Fig. 3(b)) further highlighted that CTR (cucumber) exhibited higher levels of chlorophylls (Chl a, Chl b and Chl tot), moisture content, titratable acidity and colorimetric indices compared to C and L.

Based on the clear species‐driven separation observed in the PCA, which identified cucumber and ‘Scopatizzo’ as two distinct postharvest response clusters, subsequent analyses were focused on ‘Scopatizzo’ fruits. This approach allowed a more targeted evaluation of the effects of supplemental lighting on postharvest physicochemical traits, avoiding confounding interspecific variability. Therefore, the following subsections specifically describe and discuss the postharvest behaviour of ‘Scopatizzo’ grown under natural and supplemental light conditions during cold storage.

#### Effect of cold storage on physicochemical characteristics of ‘Scopatizzo’ samples

The results of the physicochemical evaluation of unripe melon samples during cold storage are summarised in Table 4. Significant differences in moisture content were detected over the 17 days of cold storage, whereas no significant differences were observed between light treatments or for the interaction between light treatments and storage time. The observed moisture reduction, amounting to approximately 1 g 100 g^−1^ FW, can be attributed to natural transpiration or dehydration processes, which lead to a gradual loss of water during shelf‐life, in agreement with previous studies.^36^ Similarly, significant changes in titratable acidity were observed during cold storage, while no significant differences were detected between light treatments or for the light treatment × storage time interaction (Table 4). This increase can be ascribed to postharvest metabolic processes, including sugar degradation and the consequent production of organic acids. Notably, pH values exhibited a clear inverse relationship with titratable acidity; as acidity increased, a progressive decrease in pH was observed in both samples during storage. This behaviour is typical of plant matrices undergoing ripening and senescence, where oxidative, fermentative and hydrolytic reactions generate organic acids that contribute to tissue acidification. These parameters represent key indicators of product quality and stability during storage, as they allow monitoring of the chemical and microbiological evolution of the fruit.^37^ Conversely, the absence of differences between light treatments suggests that ‘Scopatizzo’ may respond differently to LED supplementation compared with other vegetable products. In this regard, Silva *et al*.^37^ reported that LED lighting can modulate metabolic processes in Barbados gooseberry, such as organic acid synthesis and intercellular pH regulation, thereby influencing product chemical composition. Similarly, Demir *et al*.^38^ observed that exposure to LED lighting with specific wavelengths increased titratable acidity in broccoli and cabbage microgreens, highlighting the active role of light in regulating the nutritional and functional quality of plants.

**Table 4:** Physicochemical properties of ‘Scopatizzo’ (*Cucumis melo* L.) grown under natural light (C) and artificial light (L) during refrigerated storage at 4 °C

	Moisture	Titratable acidity	pH	WI, pulp	WI, skin	Firmness	Springiness	Chewiness
	g 100 g^−1^ FW	g 100 g^−1^ FW				N		N
Treatment								
C	95.41 ± 0.39	0.081 ± 0.016	6.12 ± 0.09	67.74 ± 2.89	40.57 ± 1.68	15.92 ± 0.89	0.42 ± 0.04	2.68 ± 0.36
L	95.11 ± 0.44	0.078 ± 0.014	6.12 ± 0.09	66.66 ± 2.60	43.28 ± 1.06	17.84 ± 0.62	0.43 ± 0.03	2.70 ± 0.36
Time of storage								
T0	95.74 ± 0.19 a	0.068 ± 0.009 b	6.25 ± 0.02 a	63.63 ± 0.78 e	42.81 ± 1.31 a	17.65 ± 0.94 a	0.46 ± 0.02 a	2.79 ± 0.30 a
T1	95.39 ± 0.07 ab	0.064 ± 0.008 b	6.17 ± 0.04 b	65.28 ± 0.86 d	40.64 ± 2.86 b	17.07 ± 0.60 ab	0.47 ± 0.02 a	3.02 ± 0.29 a
T2	95.21 ± 0.52 b	0.084 ± 0.011 a	6.10 ± 0.05 c	68.34 ± 1.87 c	41.64 ± 2.19 ab	16.40 ± 1.29 b	0.42 ± 0.01 b	2.63 ± 0.20 ab
T3	95.20 ± 0.27 b	0.083 ± 0.011 a	6.03 ± 0.05 c	70.66 ± 0.79 a	41.58 ± 1.53 ab	16.55 ± 1.19 b	0.39 ± 0.01 b	2.71 ± 0.31 ab
T4	94.74 ± 0.29 c	0.084 ± 0.013 a	6.06 ± 0.05 c	68.95 ± 0.80 b	42.96 ± 0.73 a	16.68 ± 1.82 b	0.39 ± 0.02 b	2.30 ± 0.32 b
Significance								
Treatment	ns	ns	ns	**	***	***	ns	ns
Time of storage	***	***	***	***	**	**	***	**
Treatment × time of storage	ns	ns	ns	ns	*	**	ns	*

**Figure 4:**
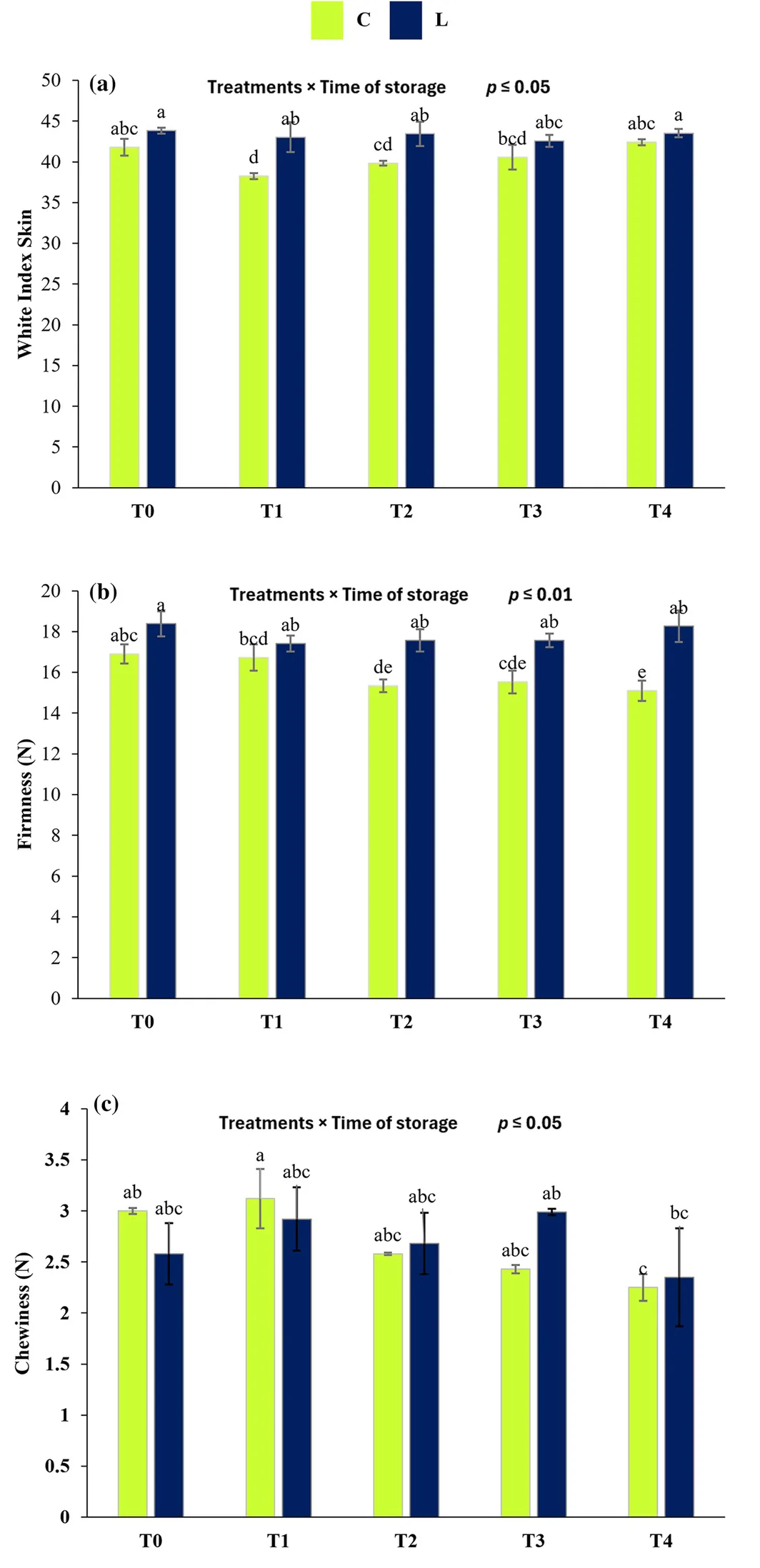
Variation of skin WI (a), firmness (b) and chewiness (c) of ‘Scopatizzo’ (*Cucumis melo* L.) fruits grown under natural light (C) and artificial light (L) during refrigerated storage at 4 °C. The same lowercase letters indicate that the mean values ± SD are not significantly different (*P* = 0.05). T0 = beginning of storage; T1 = 4 days of refrigerated storage; T2 = 8 days of refrigerated storage; T3 = 12 days of refrigerated storage; T4 = 17 days of refrigerated storage.

Different letters in each column mean a significant differences according to Tukey test. Significance: *P ≤ 0.05, **P ≤ 0.01, ***P ≤ 0.001; ns, not significant. T0 = beginning of storage; T1 = 4 days of refrigerated storage; T2 = 8 days of refrigerated storage; T3 = 12 days of refrigerated storage; T4 = 17 days of refrigerated storage. Values are mean ± SD of the main factors: Treatment (*n* = 3) and Time of storage (*n* = 6). When the interaction effect (Treatment × Time of storage) was significant, the corresponding results are reported in Fig. 4.

The analysis of colorimetric parameters highlighted the trend of the WI, which quantifies colour lightness based on the three CIELAB coordinates (L*, a*, b*). The WI measured in the pulp of fruits grown under LED lighting was approximately one unit lower than that of fruits grown without light supplementation. At the same time, pulp WI gradually increased during cold storage up to day 12, followed by a decrease on day 17 of storage (Table 4). These results suggest that exposure to low temperature may induce an increase in tissue lightness, likely associated with alterations in pigment composition.^39^ With respect to skin WI, a significant interaction between light treatments and storage time was observed (Table 4). In C fruits (grown under natural light), skin WI initially decreased slightly up to T1 (38.26), then increased to 42.40 at the end of storage (T4), indicating moderate yellowing followed by surface lightening due to chlorophyll degradation (Fig. 3(a)). Conversely, in fruits grown under LED lighting (L), skin WI remained stable throughout the storage period (Fig. 3(a)). This observation in LED‐grown fruits suggests a more balanced colour transition during cold storage, likely attributable to enhanced antioxidant protection and greater pigment stability.^30^

With respect to firmness, a significant interaction between light treatments and storage time was observed (Table 4). In C fruits, firmness decreased as of T2, reaching a value of 15.10 N at the end of storage, while the L fruits maintained relatively constant firmness values (17–18 N) throughout the 17 days (Fig. 4(c)). Significant differences in springiness were detected over the 17 days of cold storage, whereas no significant differences were observed between light treatments and for the interaction between light treatments and storage time (Table 4). Notably, the springiness value decreased starting from T2 and remained constant until the end of the storage (T4). With respect to chewiness, a significant interaction between light treatments and storage time was observed (Table 4). At T0 both treatments showed comparable values (*ca* 2.6–3.0). In fruits grown under natural light (C), chewiness value at T4 was lower than at the initial storage period (T0 and T1), while the L fruits maintained relatively constant chewiness values throughout the 17 days (Fig. 3(b)).

These results suggest that preharvest light supplementation may reduce cell wall degradation, likely associated with lower enzymatic activity and enhanced tissue integrity under cold stress conditions.

The loss of firmness in fruits and vegetables during postharvest storage is closely related to the degradation of pectin in cell walls. This degradation is associated with the enzymatic activity of pectin esterase, polygalacturonase, pectin lyase and *β*‐galactosidase. Pectin esterase de‐esterifies pectin, converting it into pectic acid, which is then hydrolysed by polygalacturonase; pectin lyase breaks glycosidic bonds, while *β*‐galactosidase increases pectin solubility. Overall, these enzymatic activities compromise the integrity of the cell wall, leading to the enlargement of intercellular spaces and tissue softening,^40^ ultimately resulting in the loss of firmness and quality in postharvest fruits.^41^

Other authors^2^, ^42^ have reported that the mechanical stability of fruits is associated with delayed cell wall loosening and reduced water loss as a consequence of cellular metabolism modulated by controlled lighting conditions.

Therefore, LED treatment may represent a promising strategy to mitigate the reduction of mechanical stability in fruits during cold storage. Supporting this, Wang *et al*.^43^ reported that the exposure of white ginger to blue LED light better preserved firmness due to a greater protopectin accumulation and a reduction in soluble pectin, which help maintain cell cohesion, as well as the inhibition of enzymes that degrade the cell wall, slowing down the softening process.

#### Effect of cold storage on chemical characteristics of ‘Scopatizzo’ samples

**Figure 5:**
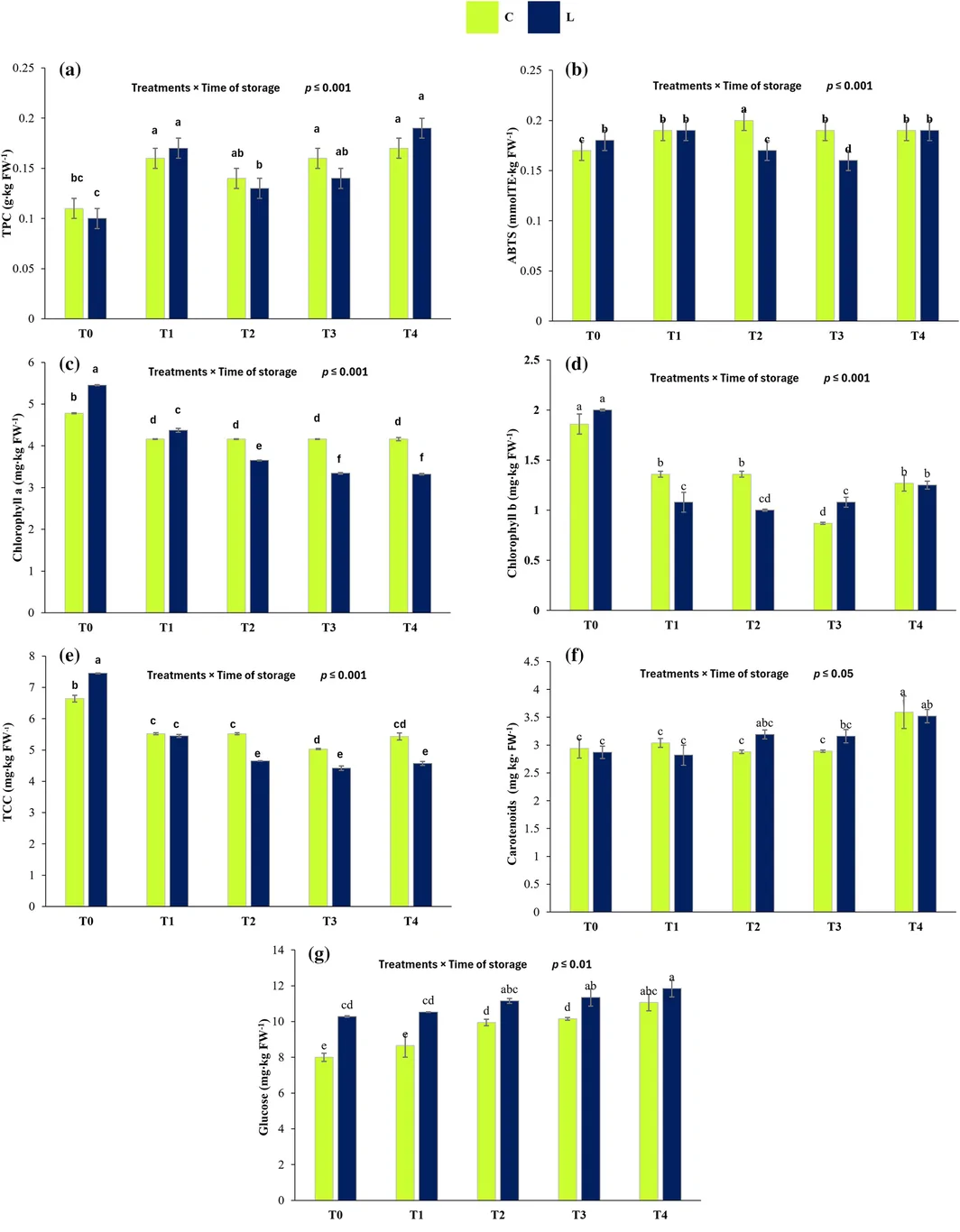
Variation of TPC (a), ABTS (b), chlorophyll a (c), chlorophyll b (d), TCC (total chlorophyll content) (e), carotenoids (f) and glucose (g) of ‘Scopatizzo’ (*Cucumis melo* L.) fruits harvested under artificial light (L) and natural light (C) conditions during refrigerated storage at 4 °C. The same lowercase letters indicate that the mean values ± SD are not significantly different (*P* = 0.05). T0 = beginning of storage; T1 = 4 days of refrigerated storage; T2 = 8 days of refrigerated storage; T3 = 12 days of refrigerated storage; T4 = 17 days of refrigerated storage.

The effects of cold storage on the quality traits of ‘Scopatizzo’ fruits were evaluated in terms of chemical compounds such as polyphenols, chlorophylls, carotenoids and sugars, as well as their antioxidant activity, and are reported in Table 5. With the exception of fructose content, a significant interaction between light treatment and storage time was observed for all measured parameters (Table 5). With respect to TPC, the lowest TPC was observed in ‘Scopatizzo’ fruits grown under LED lighting at T0 (0.10 mg g^−1^ FW; Fig. 5(a)). This value was approximately 31% lower than the average TPC recorded during storage (T1–T4), which was 0.16 mg g^−1^ FW (Fig. 5(a)). Our results are in line with those previously reported by Palmitessa *et al*.,^2^ who did not observe differences in TPC in immature melon fruits grown with or without supplemental lighting. However, an interesting aspect emerging from our study is that storage at 4 °C consistently increased TPC, regardless of light supplementation during cultivation. This behaviour is consistent with the stimulation of phenylalanine ammonia lyase and other enzymes involved in secondary metabolism as a defence mechanism against chilling stress.^29^, ^32^, ^44^, ^45^

**Table 5:** Chemical composition of ‘Scopatizzo’ (*Cucumis melo* L.) grown under natural light (C) and artificial light (L) during refrigerated storage at 4 °C

	Total phenolic content	ABTS	Chlorophyll a	Chlorophyll b	Total chlorophyll content	Carotenoids	Glucose	Fructose
	mg g^−1^ FW	mmol TE g^−1^ FW	mg kg^−1^ FW	mg kg^−1^ FW	mg kg^−1^ FW	mg kg^−1^ FW	mg g^−1^ FW	mg g^−1^ FW
Treatment								
C	0.15 ± 0.02	0.19 ± 0.01	4.40 ± 0.31	1.35 ± 0.33	5.75 ± 0.47	3.07 ± 0.31	9.56 ± 1.18	10.77 ± 1.86
L	0.15 ± 0.03	0.18 ± 0.01	4.05 ± 0.84	1.28 ± 0.38	5.33 ± 1.21	3.11 ± 0.28	11.03 ± 0.63	12.31 ± 1.58
Time of storage								
T0	0.10 ± 0.01 e	0.18 ± 0.01 b	5.11 ± 0.37 a	1.93 ± 0.10 a	7.04 ± 0.45 a	2.90 ± 0.13 b	9.14 ± 1.26 c	9.48 ± 1.15 d
T1	0.16 ± 0.01 b	0.19 ± 0.01 a	3.90 ± 0.28 d	1.22 ± 0.17 b	5.12 ± 0.43 c	2.93 ± 0.17 b	9.59 ± 1.11 c	10.47 ± 1.06 c
T2	0.14 ± 0.01 d	0.18 ± 0.02 a	3.74 ± 0.44 e	1.19 ± 0.20 b	4.93 ± 0.64 d	3.06 ± 0.18 b	10.63 ± 0.68 b	11.44 ± 0.74 c
T3	0.15 ± 0.01 c	0.17 ± 0.02 b	4.05 ± 0.80 c	0.98 ± 0.11 c	5.03 ± 0.69 cd	3.03 ± 0.16 b	10.75 ± 0.72 b	12.58 ± 0.87 b
T4	0.18 ± 0.01 a	0.19 ± 0.01 a	4.31 ± 0.18 b	1.26 ± 0.06 b	5.58 ± 0.18 b	3.55 ± 0.20 a	11.45 ± 0.59 a	14.03 ± 1.00 a
Significance								
Treatment	ns	***	***	**	***	ns	***	***
Time of storage	***	***	***	***	***	***	***	***
Treatments × time of storage	***	***	***	***	***	*	**	ns

Different letters in each column mean significant differences according to Tukey test. Significance: *P ≤ 0.05, **P ≤ 0.01, ***P ≤ 0.001; ns, not significant. T0 = beginning of storage; T1 = 4 days of refrigerated storage; T2 = 8 days of refrigerated storage; T3 = 12 days of refrigerated storage; T4 = 17 days of refrigerated storage. Values are mean ± SD of the main factors: Treatment (*n* = 3) and Time of storage (*n* = 6). When the interaction effect (Treatment × Time of storage) was significant, the corresponding results are reported in Fig. 5.

With respect to ABTS, at harvest (T0), fruits from plants grown under light supplementation showed an ABTS value 6% higher than that of C fruits. However, no differences between light treatments were observed at either T1 or T4. Overall, while storage at 4 °C had a positive effect on ABTS values for C fruits, the trend observed in L fruits did not show an increase but rather a decrease during the intermediate storage period (T2 and T3) (Fig. 5(b)). The higher ABTS value of L fruits at T0 may be due to a higher content of total chlorophyll (Fig. 5(e)) and molecules with antioxidant activity, such as antheraxanthin, lutein and *β*‐carotene. This in agreement with a previous report of Palmitessa *et al*.^2^ for immature melon fruits grown under supplemental LED lighting. On the other hand, the slight differences in ABTS values observed between the two treatments during the intermediate storage period (T2 and T3) may be due to the complex interaction of the various antioxidant components present in the fruits (Fig. 5).

Instead, chlorophyll a content was 14% and 8% higher in ‘Scopatizzo’ fruits grown under artificial light compared with those grown under natural light at T0 and T1, respectively (Fig. 5(c)). In contrast, at T2, T3 and T4, chlorophyll a content was 12, 20 and 20% higher, respectively, in fruits grown under natural light than in those grown under artificial light (Fig. 5(c)). This pattern indicates a faster decline of chlorophyll a during storage in fruits produced under artificial lighting compared with the control. Similarly, a decrease in chlorophyll b was observed during storage at 4 °C (Fig. 5(d)). In this case, no clear trend of faster chlorophyll b degradation was observed in either treatment. At T0, the mean chlorophyll b content was 1.90 mg kg^−1^ FW in fruits harvested under both light conditions (Fig. 5(d)). At T1 and T2, chlorophyll b content was approximately 28% lower in LED‐grown fruits compared with C (Fig. 5(d)). However, unlike what was observed for chlorophyll a, at T3 chlorophyll b content was about 25% higher in ‘Scopatizzo’ fruits grown under artificial light than in those grown under natural light (Fig. 5(d)). At T4, the mean chlorophyll b content was 1.26 mg kg^−1^ FW in both treatments, corresponding to an overall reduction of approximately 70% compared with T0 values (Fig. 5(d)). Furthermore, considering total chlorophyll content (TCC; Fig. 5(e)), at harvest (T0) ‘Scopatizzo’ fruits grown under artificial lighting showed TCC values 12% higher than those grown under natural light. At T1, both treatments exhibited a comparable mean TCC of 5.49 mg kg^−1^ FW (Fig. 5(e)). At T2, T3 and T4, fruits grown under natural light (C) showed TCC values that were 19, 14 and 19% higher, respectively, than those of fruits grown under artificial light (Fig. 5(e)). This pattern largely reflects the trend observed for chlorophyll a, which accounts for more than 70% of TCC and therefore predominantly determines the overall TCC dynamics. The degradation of chlorophyll during the postharvest of green horticultural crops is one of the main quality problems occurring during storage. In crops such as leafy vegetables, broccoli and lime, chlorophyll a degradation was thought to lead to the formation of chlorophyllide a by chlorophyllase, and pheophytin a formation by Mg removal from the porphyrin ring.^46^

The higher pigment concentrations measured at harvest in LED‐grown fruits confirm the known stimulatory effect of red–blue light on chlorophyll biosynthesis during cultivation.^47^, ^48^ However, during storage both treatments exhibited comparable degradation trends, with the stronger initial drop in LED‐grown fruits likely reflecting their larger starting chlorophyll pool rather than a fundamentally different postharvest response.

In both cases, chlorophyll loss paralleled the increase in WI of pulp and skin, supporting the association between pigment degradation and tissue lightning under cold‐storage conditions. This alignment indicates that preharvest lighting influenced the initial pigment content, whereas the subsequent evolution of colour was mainly driven by storage‐related physiological processes.

As reported by Liu *et al*.,^30^ chlorophyll degradation is mediated by specific enzymes, whose activity is often induced by abiotic stresses (including low temperatures) that accelerate senescence and pigment degradation processes. In line with these findings, Pulela *et al*.^49^ confirmed that chlorophyll concentrations tend to decrease during cold storage due to degradation of photosynthetic pigments mediated by physiological stress.

Carotenoid dynamics during storage showed a pattern that contrasted with that of chlorophylls (Fig. 5(f)). At harvest (T0), both treatments exhibited similar carotenoid concentrations (2.90 mg kg^−1^ FW). Across storage, carotenoid levels remained relatively stable from T0 to T3 and then increased significantly at T4, reaching about 3.55 mg kg^−1^ FW, regardless of the preharvest light regime (Fig. 5(f)). This late rise in carotenoids is consistent with their role as photoprotective and antioxidant pigments,^42^ which can accumulate in response to oxidative conditions associated with chlorophyll degradation and tissue senescence.^50^ In line with this interpretation, the progressive decline in total chlorophylls, together with the increase in carotenoids and pulp WI, supports the view that cold storage triggered coordinated pigment remodelling, with carotenoids contributing to the mitigation of oxidative stress and to the stabilisation of membrane structures, rather than revealing a clear differential response between natural‐light‐grown and LED‐grown fruits.

For glucose contents, a significant interaction between light treatment and storage time was observed (Fig. 5(g)). In C fruits, glucose content increased from 8.0 at T0 to 11.1 mg g^−1^ at T4, whereas in L fruits it rose from 10.3 to 11.8 mg g^−1^ at T4 (Fig. 5(g)). The higher initial glucose content observed in L fruits (T0) suggests a more efficient carbohydrate synthesis, attributable to an enhanced photosynthetic process, likely due to a greater accumulation of photoassimilates during cultivation under artificial lighting conditions. For fructose, the mean concentrations in artificial‐light‐grown fruits were 14% more compared with natural‐light‐grown ones (C) (Table 5). In addition, the average fructose content increased from 10.8 at T0 to 12.3 mg g^−1^ at T4 (Table 5). These results reinforce the hypothesis on the influence of preharvest light conditions on sugar metabolism, consistent with previous observations in immature *Cucumis melo*, where LED illumination enhanced carbohydrate biosynthesis and storage in the fruit tissues.^2^, ^14^ The observed rise in sugar concentration during refrigerated storage is likely attributable to partial water loss during storage (Table 4), which may contribute to an apparent increase in sugar concentration. Similar trends have been reported in both mature and immature melons and related cucurbit fruits stored under refrigeration, where sugar levels remained stable or showed slight increases due to concentration effects rather than active biosynthesis.^49^ In addition, the observed rise in glucose concentration during refrigerated storage could also reflect residual metabolic activity, contributing to osmotic adjustment and maintenance of tissue turgidity.^2^

## CONCLUSIONS

This study provides the first evidence on the postharvest behaviour of whole, unripe fruits of the local melon landrace ‘Scopatizzo’ and shows that preharvest red–blue LED lighting can modulate fruit quality during cold storage. LED supplementation increased yield at harvest and improved firmness retention over 17 days of postharvest storage at 4 °C, without affecting mineral composition. The main effect concerned textural stability, as LED‐grown fruits maintained higher firmness and chewiness, while changes in moisture, pH and titratable acidity were mainly driven by storage time. Although LED‐exposed fruits exhibited higher initial chlorophyll and sugar levels, phenolics and carotenoids increased during storage in both treatments, indicating a predominant effect of cold‐induced responses. Overall, the results support the integration of LED lighting and soilless cultivation to enhance productivity and postharvest performance of local melon landraces, though further studies are needed to clarify underlying biochemical mechanisms and assess commercial shelf‐life under broader storage conditions. These findings also confirm the potential of ‘Scopatizzo’ as a viable alternative to cucumber in the fresh market. In fact, it exhibited a postharvest behaviour comparable to that reported for cucumber, suggesting a potential marketability of up to 17 days under the tested storage conditions.

## AUTHOR CONTRIBUTIONS

Marica Troilo: Writing original draft, visualisation, methodology, investigation, formal analysis, data curation, conceptualisation. Onofrio Davide Palmitessa: Writing original draft, visualisation, methodology, investigation, formal analysis, data curation, conceptualisation. Massimiliano Renna: Writing – review and editing, visualisation, supervision, methodology, conceptualisation. Davide De Angelis: Writing – review and editing, visualisation, methodology, formal analysis, data curation, conceptualisation. Carlo Porfido: Writing original draft, methodology, data curation. Roberto Terzano: Writing – review and editing, supervision, resources, conceptualisation. Francesco Caponio: Writing – review and editing, visualisation, validation, supervision, methodology, funding acquisition. Pietro Santamaria: Writing – review and editing, visualisation, validation, supervision, project administration, methodology, investigation, funding acquisition.

## FUNDING INFORMATION

This research was funded by the Agritech National Research Center and received funding from the European Union Next‐Generation EU (PIANO NAZIONALE DI RIPRESA E RESILIENZA (PNRR) – MISSIONE 4 COMPONENTE 2, INVESTIMENTO 1.4 – D.D. 1032 17/06/2022, CN00000022). This paper reflects only the authors' views and opinions, neither the European Union nor the European Commission can be considered responsible for them.

## CONFLICT OF INTEREST

The authors declare that they have no known competing financial interests or personal relationships that could have appeared to influence the work reported in this paper.

## Data Availability

The data that support the findings of this study are available from the corresponding author upon reasonable request.
